# Prognostic value of red cell distribution width (RDW) in colorectal cancer. Results from a single-center cohort on 591 patients

**DOI:** 10.1038/s41598-020-57721-4

**Published:** 2020-01-23

**Authors:** Corrado Pedrazzani, Marzia Tripepi, Giulia Turri, Eduardo Fernandes, Giovanni Scotton, Simone Conci, Tommaso Campagnaro, Andrea Ruzzenente, Alfredo Guglielmi

**Affiliations:** 1 Division of General and Hepatobiliary Surgery, Department of Surgical Sciences, Dentistry, Gynecology and Pediatrics, University of Verona, Verona, Italy; 2 Division of Minimally Invasive, General and Robotic Surgery, University of Illinois at Chicago, Chicago, US

**Keywords:** Colorectal cancer, Prognostic markers

## Abstract

Increasing evidence advocates the prognostic role of RDW in various tumours. We analysed 591 patients to assess whether RDW is a prognostic factor for overall (OS) and cancer-related survival (CRS) for patients with colorectal cancer (CRC). The data were retrieved from a retrospective database. The optimal cut-off value for RDW was set at 14.1%; accordingly, two groups were considered: those with a value equal or lower than 14.1% (L-RDW), and those with a value higher than 14.1% (H-RDW). The mean value of RDW rose from pT1 to pT4 tumours. H-RDW correlated with age above the mean, colonic location of the lesion, pT and TNM stage. Finally, H-RDW was significantly associated with the intent of surgery: almost 50% of patients who underwent a non-curative resection presented H-RDW, compared to 19.3% in R0 resections. OS was significantly lower in patients with H-RDW. CRS was similar in the two groups. Stratifying patients according to TNM stage worse OS was associated with H-RDW only in early stages, whereas there was no difference for stages II-IV. Multivariate analysis confirmed that H-RDW was not an independent prognostic factor. Although H-RDW correlated with some negative clinical-pathological factors, it did not seem to independently influence OS and CRS.

## Introduction

Colorectal cancer (CRC) is the third most common cancer worldwide, with more than 1 million new cases and 600.000 deaths per year^1^.

Several biochemical markers related to the inflammatory processes that accompanies this malignancy have recently surged as diagnostic and prognostic tools^2–4^.

Beside classical ‘inflammatory related’ markers such as acute phase proteins (CRP and globulins), also parameters that reflect changes in certain bone marrow lineages such as PLR and NLR have been described^2,5^.

Amongst these, red blood cell distribution width (RDW) is a parameter that reflects the size heterogeneity of red blood cells and is normally used to differentiate various types of anemia^6^.

More recently, RDW has surged as a biochemical marker in several chronic inflammatory and cardiovascular disease^7–9^.

Recent reports have shown how it can be used as a prognostic marker in various cancer such as, lung, liver, esophago-gastric and breast^10–15^. RDW has been studied as a potential prognostic marker also in CRC. In the context of this malignancy, however, its role remains unclear, as reports so far published have shown inconsistent results.

The aim of this retrospective study was to evaluate the prognostic value of red blood cell distribution width in a large cohort of patients undergoing surgery for colorectal cancer.

## Results

During the study period, 1347 patients underwent surgery for CRC. Among these, 591 met the inclusion criteria and were included in the final analysis.

The pre-operative mean RDW value (±SD) was ±15.2% (±3.2%) and the median value (range) was 14.1% (11.6–31.8%).

The correlations of RDW with clinico-pathological variables are shown in Table 1. Both the mean RDW value and the percentage of H-RDW cases were higher in patients with increased age, colon cancer (vs rectal), locally advanced tumors and higher TNM stages. Furthermore, H-RDW was more frequently observed in non-curative resections. No association was present with gender, nodal involvement, presence of systemic metastasis, histological type and tumor grading.

**Table 1:** Correlations between RDW and main clinicopathological variables for the 591 patients under study.

Data	Pts.	Mean (±SD) RDW	p value	H-RDW	p value
Age			<**0.001**		<**0.001**
≤67.5 years	296	14.7 (±2.8)		119 (40.2%)	
>67.5 years	295	15.7 (±3.4)		164 (55.6%)	
Gender			0.821		0.108
Male	349	15.2 (±2.9)		175 (50.1%)	
Female	242	15.2 (±3.5)		108 (44.6%)	
Tumor location			<**0.001**		**0.012**
Colon	444	15.5 (±3.4)		225 (50.7%)	
Rectum	147	14.3 (±1.9)		58 (39.5%)	
Intent of surgery			0.286		**0.001**
R0 resection	504	15.3 (±3.2)		21 (19.3%)	
R1-2/No resection	87	14.9 (±2.8)		42 (48.3%)	
Depth of invasion (pT)			**0.017**		**0.037**
pTis	62	14.4 (±2.2)		26 (41.9%)	
pT1	91	14.9 (±2.8)		42 (46.2%)	
pT2	69	14.7 (±2.8)		25 (36.2%)	
pT3	227	15.5 (±3.3)		116 (51.1%)	
pT4	137	15.7 (±3.6)		75 (54.7%)	
Nodal involvement (pN)			0.856		0.332
pN0	350	15.3 (±3.4)		160 (45.7%)	
pN1	152	15.1 (±2.8)		77 (50.7%)	
pN2	84	15.3 (±2.9)		45 (53.6%)	
Systemic metastasis (M)			0.243		0.212
M0	472	15.3 (±3.4)		225 (47.7%%)	
M1a	84	15 (±2.2)		46 (54.8%)	
M1b	30	14.4 (±2.1)		11 (36.7%)	
TNM stage			**0.002**		**0.049**
Stage 0-I	175	14.7 (±2.8)		69 (39.4%)	
Stage II	155	15.9 (±4)		80 (51.6%)	
Stage III	142	15.5 (±3.2)		76 (53.5%)	
Stage IV	114	14.8 (±2.2)		57 (50%)	
Histological type			0.594		0.267
Adenocarcinoma	495	15.2 (±3.3)		230 (46.5%)	
Mucinous	82	15.5 (±2.8)		46 (56.1%)	
Others	14	14.8 (±1.8)		7 (50%)	
Tumor grading			0.839		0.134
G1-G2	533	15.4 (±3.6)		217 (48.8%)	
G3	58	15.5 (±3.1)		28 (58.3%)	

SD: standard deviation.

Overall and cancer-related survival rates in relationship to the main clinicopathological variables are reported in Table 2. Age, intent of surgery, depth of tumour invasion (pT), node involvement (pN), metastatic disease (M), TNM stage and tumor grading were confirmed to be significant predictors of overall and cancer-related survival. Females showed a better overall survival rate compared to males but a comparable cancer-related survival.

**Table 2:** Kaplan-Meier estimates of survival probability according to main clinical-pathological variables for the 591 patients under study.

Data	Pts.	5-yr overall survival	p value	5-yr cancer-related survival	p value
Age			<**0.001**		**0.011**
≤67.5 years	296	79.7%		83.2%	
>67.5 years	295	67.7%		75.2%	
Gender			**0.019**		0.112
Male	349	71%		77.7%	
Female	242	76.8%		81.6%	
Tumor location			0.917		0.777
Colon	444	73.7%		79.6%	
Rectum	38	72.7%		78.3%	
Intent of surgery			<**0.001**		<**0.001**
R0 resection	504	79.6%		85.9%	
R1-2/No resection	87	38%		39.7%	
Depth of invasion (pT)			<**0.001**		<**0.001**
pTis	62	91.4%		100%	
pT1	91	85.1%		89.6%	
pT2	69	84%		89.6%	
pT3	227	75.8%		76.2%	
pT4	137	51.5%		57.6%	
Nodal involvement (pN)			<**0.001**		<**0.001**
pN0	350	86.1%		92.4%	
pN1	152	63.9%		68.1%	
pN2	84	40.2%		47.8%	
Systemic metastasis (M)			<**0.001**		<**0.001**
M0	472	81.3%		87.9%	
M1a	84	46.6%		48.5%	
M1b	30	—		—	
TNM stage			<**0.001**		<**0.001**
Stage 0-I	175	92.6%		97.4%	
Stage II	155	80.8%		89.8%	
Stage III	142	68.5%		73.8%	
Stage IV	114	42.1%		43.8%	
Histological type			0.066		0.218
Adenocarcinoma	495	74.6%		80%	
Mucinous	82	70.3%		77.2%	
Others	14	42.9%		69.6%	
Tumor grading			<**0.001**		<**0.001**
G1-G2	533	76.1%		81.7%	
G3	58	41.7%		49.5%	

SD: standard deviation.

**Figure 1:**
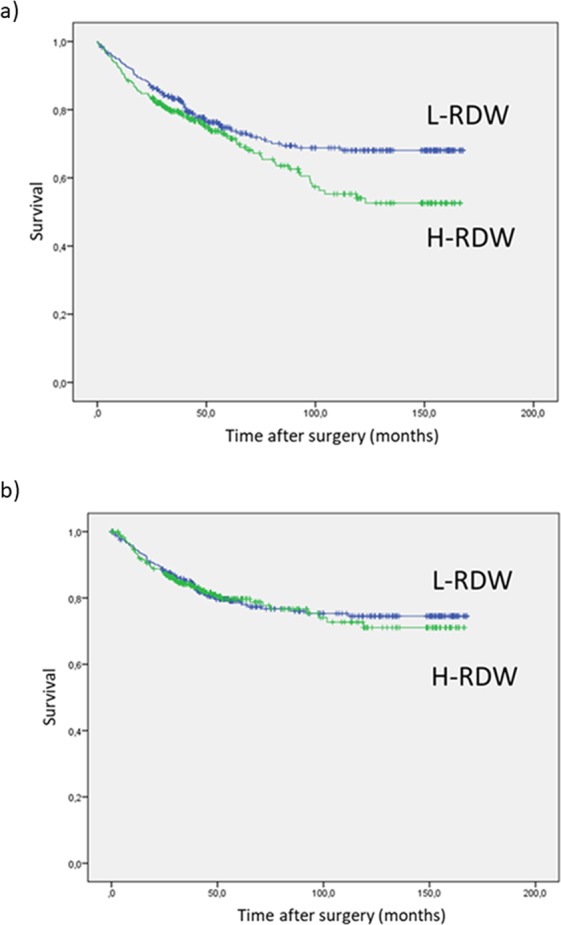
(**a**) Overall survival stratified by RDW (p = 0.043); (**b**) Cancer-related survival stratified by RDW (p = 0.775).

Figure 1 shows the overall and cancer-related survival according to RDW value. H-RDW group demonstrated a significantly lower long-term survival compared to L-RDW group (p = 0.043). Interestingly, overall survival at 5-years was similar between the two groups (L-RDW: 74.7% vs. H-RDW: 72.3%), but diverted over time reaching a significant difference at 10-years (L-RDW: 68.1% vs. H-RDW: 54%; p = 0.043) (Fig. 1a). This phenomenon was not observed when analysing the cancer-related survival (Fig. 1b), which remained similar throughout the study period.

In the Cox regression model, both considering overall and cancer-related survivals, RDW controlled for age, gender, tumor location, intent of surgery and TNM stage, did not prove to be an independent predictor of prognosis (Table 3).

**Table 3:** Multivariable survival analysis including RDW for the 591 patients under study.

Data	Overall survival: HR (95% CI)	p value	Cancer related survival: HR (95% CI)	p value
Age		<**0.001**		<**0.001**
≤67.5 years	—		—	
>67.5 years	2.62 (1.88–3.64)		2.22 (1.52–3.24)	
Gender		0.521		0.903
Female	—		—	
Male	1.11 (0.8–1.54)		0.98 (0.67–1.43)	
Tumor location		0.376		0.353
Colon	—		—	
Rectum	0.85 (0.6–1.21)		0.82 (0.55–1.24)	
Intent of surgery		<**0.001**		<**0.001**
R0 resection	—		—	
R1-2/No resection	1.75 (1.36–2.26)		1.72 (1.31–2.26)	
TNM stage		<**0.001**		<**0.001**
Stage 0-I	—		—	
Stage II	1.33 (0.78–2.3)		1.63 (0.7–3.78)	
Stage III	3.04 (1.84–5)		5.78 (2.77–12.1)	
Stage IV	4.94 (2.76–8.88)		11.48 (5.18–25.42)	
Red cell distribution width		0.312		0.632
L-RDW	—		—	
H-RDW	1.17 (0.86–1.6)		0.915 (0.64–1.32)	

^a^Values in parentheses are 95% confidence intervals. Hazard ratio and P values were derived from Cox regression analysis, controlling for all other variables.

**Figure 2:**
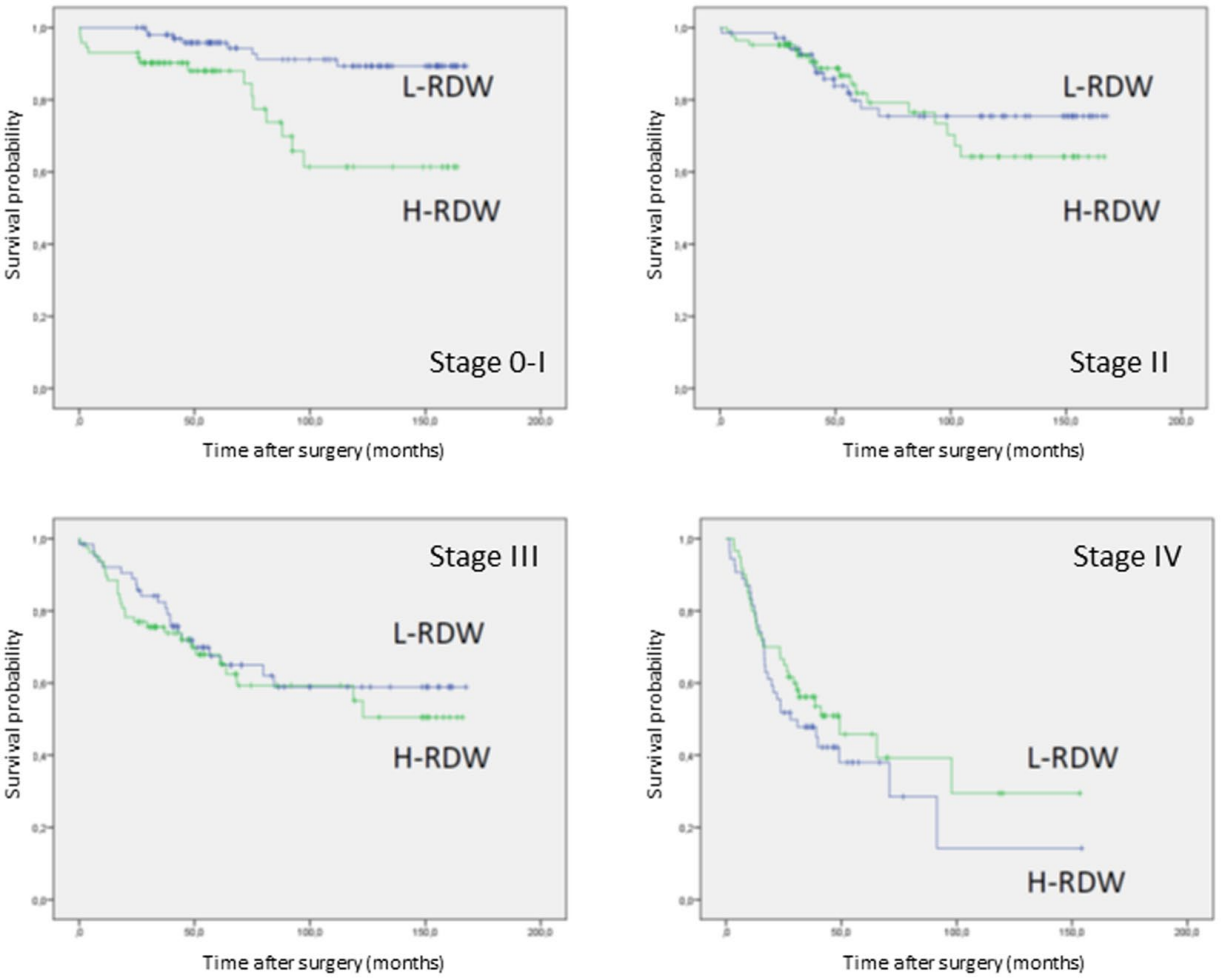
Overall survival for TNM Stages 0-I (p = 0.001), II (p = 0.536), III (p = 0.523) and IV (p = 0.309) stratified by RDW.

**Figure 3:**
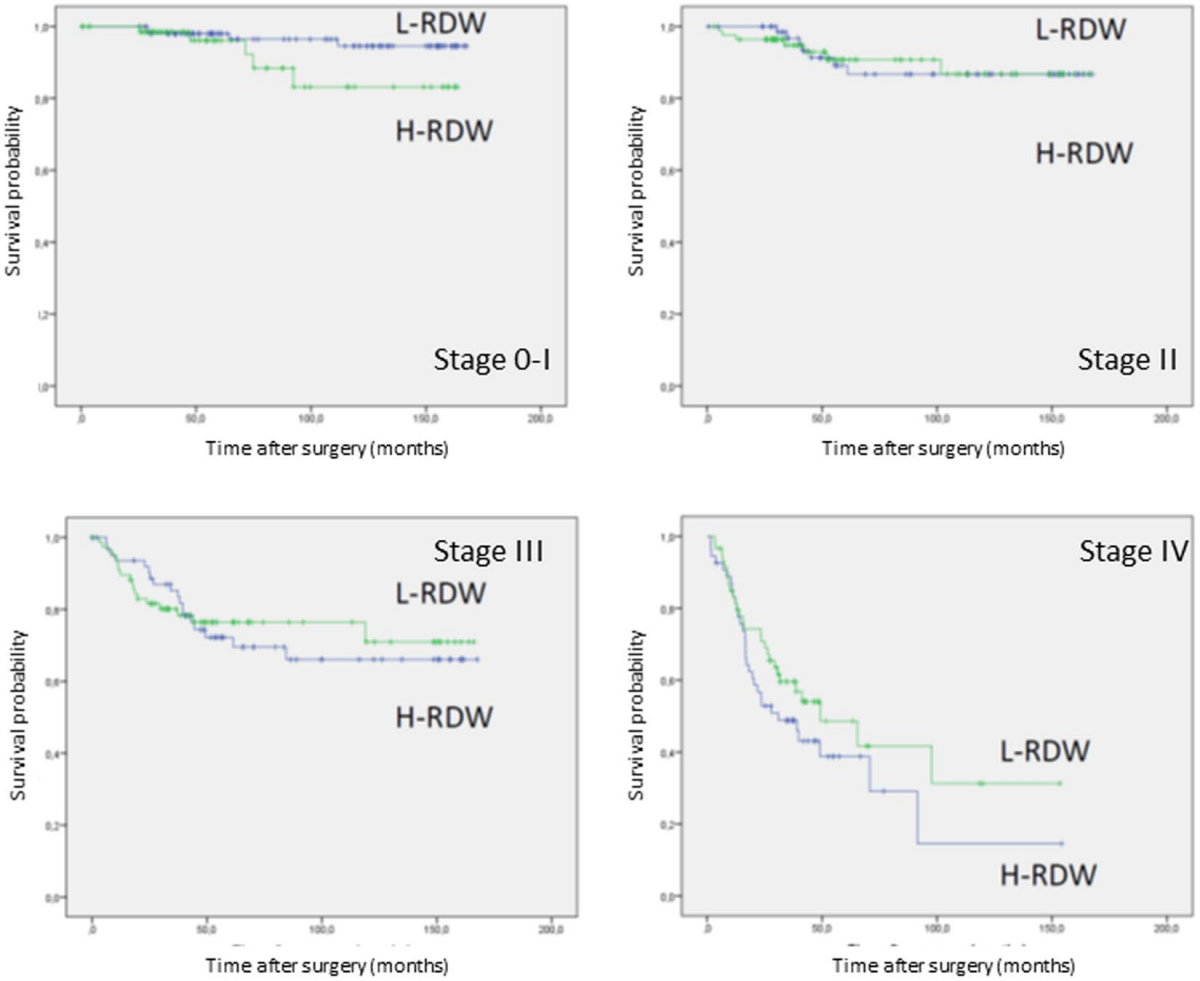
Cancer-related survival for Stages 0-I (p = 0.104), II (p = 0.996), III (p = 0.810) and IV (p = 0.201) stratified by RDW.

To further investigate the role of RDW in influencing overall mortality, survival rates were stratified according to TNM stage (Fig. 2). Based on staging, higher level of RDW were associated to decreased survival only in stage I cancers. (p = 0.001). H-RDW did not seem to impact survival in stages II, III and IV. When considering cancer-related survival, no differences were observed between high and low RDW levels (Fig. 3).

## Discussion

RDW is an indicator of heterogeneity of red cell volume and it has been used in the diagnosis and differentiation of several types of anemias as well as cardiovascular and infectious diseases^16–18^.

More recently, increased RDW values have been found to be a negative predictors of survival in several types of malignancies^10^. Some authors have reported correlations between H-RDW and decreased survival in lung^1,2^, gastric^13^, esophageal^14^, hepatocellular cancers^12,19^, and breast cancer^15^. A similar correlation also seems to apply to colorectal cancers^20,21^.

With the present study we intended to investigate whether high values of RDW correlated with poorer survival in colorectal cancer. As a corollary, we also wanted to evaluate the relationship between RDW and the main known prognostic variables related to this malignancy.

Our main finding is that patients with H-RDW have a lower 10-year overall survival compared to the ones with L-RDW. Interestingly, such difference is not visible at an earlier stage of the follow-up (5-years). On the other hand, patients with H-RDW levels did not show to have shorter cancer-related survival.

Another finding of our study is that only patients with early stage CRC (stage I) appear to have a worse survival when presenting with an elevated-RDW, which did not appear to have any impact on overall survival in more advanced stages.

In addition, the mean RDW value and the percentage of patients with H-RDW were found to be higher both in subjects with greater tumour depth of invasion (T stage) and more advanced overall TNM stages (p = 0.049). Also increased age and colon as opposed to rectal cancer were found to correlate with higher RDW values. Furthermore, H-RDW was more frequently observed in non-curative resections. No association was present between H-RDW and gender, nodal involvement, presence of systemic metastasis, histological type and tumor grading.

On first sight, it may appear that RDW has a rather spurious association with survival in colorectal cancer. In order to interpret these findings, some careful considerations should be made about the relationship between cancer, inflammation and RDW changes.

Zhang *et al*.^21^, in a cohort of 625 patients with rectal cancer undergone curative resections without prior neoadjuvant treatment, found H-RDW to be associated with poorer overall and disease-free survival. On multivariate analysis, they also found RDW to be an independent prognostic factor of poor disease free survival. Li *et al*.^22^, in a retrospective analysis of 168 patients with colo-rectal cancer, found a linear correlation between RDW levels and both 3- and 5-years disease free and overall survival. They also found that the H-RDW patients were more likely to have serosa infiltration, nodal metastases and higher TNM stages. Song *et al*.^23^, in a retrospective study including 783 patients with CRC, demonstrated that H-RDW was associated to higher pT stages, pM stages, and tumor size, as well as CEA levels. No association was found with pN stages. In their study, they also demonstrated that RDW, combined to CEA and CA19.9, has a potential function as a biomarker for the diagnosis and prognosis of CRC. Also Yang *et al*.^20^, in a small retrospective series of 85 patients, found that patients with stage III and IV CRC disease had higher values of RDW compared to patients with stages I and II disease. RDW was also found to be higher in more advanced T stages, N stages and in the presents of metastases. Similarly, Kust *et al*.^24^, in a retrospective study of 90 patients with CRC, found that H-RDW was associated to a poorer overall survival. However, H-RDW was a negative prognostic factor only in stage II cancers.

In light of these results the questions whether RDW is a reflection of tumor bulk or cancer triggered inflammation, or whether is it the result of other factors that may promote an inflammatory *milieu* where cancer growth is facilitated, remains to be addressed.

Some of the above-mentioned studies would suggest that RDW varies in relation to the tumor burden and it is strictly related to it. Some other studies showed a weaker correlation between RDW values and tumor bulk, whereby only tumor spreading locally (T stage) rather than distally (N and M stage), seems to affect the RDW.

To explain such discrepancies an attempt could be made by considering the pathophysiology of RDW alterations. RDW is considered an inflammatory associated marker, and emerging studies suggested it might be a potential factor for predicting overall mortality in a variety of human inflammatory diseases. It is well known that inflammation is a hallmark of malignancies^25,26^.

In colorectal cancer, dysregulated inflammatory response due to the presence of either germline mutations (FAP syndrome) or gut microbiome appear to be responsible for DNA damage at the basis of CRC tumorigenesis.

Previous authors have elegantly provided a detailed description of the local immune response occurring in the presence of colorectal cancer^26^. Those studies, however, show that the strong immune response of a ‘T cell’ connotation, correlated to a better prognosis due to less frequent perineural and lympho-vascular invasion. In other words, it would be the ability of the cancer cells to ‘hide’ from T cells to determine a weaker immune response and a worse prognosis as a consequence. Colorectal cancer spread to lymph nodes and distant organs may be perpetrated by cells that have already acquired the ability to escape host T cell defense mechanisms and will not trigger a cancer-directed immune response and inflammation.

This may explain why RDW seems to better correlate with the local tumor burden (T stage), especially in its initial stages when other factors such as anemia, malnutrition, and infection do not act as confounders for RDW values. Given the link between cancer and inflammation, some authors have investigated acute phase proteins as biochemical markers in colorectal cancer. The C-reactive protein, a well-known acute phase protein, has proved to have a strong ‘dose-response’ association with colorectal cancer^27^. Other authors have found that low albumin/globulin ratios (AGR), a marker of chronic inflammation, is a significant predictor of mortality in colorectal cancer patient^28^.

Chronic inflammation, whatever the trigger, causes a myriad of molecular and cellular signaling pathways changes. Some of those changes can eventually translate into functional impairment at tissue or even organ level. Bone marrow lineages alterations have been extensively described in the presence of acute and chronic inflammation.

In the context of CRC however, it remains to be understood whether the above-mentioned inflammatory *milieu* and the molecular markers associated to it are a reflection of an environment that favors tumorigenesis or rather the direct effect of the presence of the malignancy.

If the latter was true, we should observe a consistent correlation between the tumor burden and the level of inflammation. In case of inflammatory related parameters such as neutrophil-to-lymphocytes ratio (NLR), Platelet to lymphocytes ratio (PLR) and RDW, we should notice at least a partial correlation between their values with the more extensive tumors.

As a matter of fact, this is not invariably the case. Besides the lack of correlation between H-RDW and tumor burden found in our study, our same group have previously analyzed other inflammatory associated markers such as NLR and PLR, and found the association between high levels of those markers and overall and disease free survival to be unconvincing^2^.

Another factor that may explain such variability of results is the study patient population.

The studies where RDW was found to correlate to the tumor burden are mostly from eastern centers. It is a well renown fact that eastern populations suffer less co-morbidities and are better surgical candidates. The presence of other pathologies in the western patient population may affect their ‘inflammatory’ status and interfere with the ‘dose-effect’ cancer burden-inflammation level found in eastern studies. This may signify that the overall mortality of western patients with increased RDW could be due to other factors not directly related to the colorectal malignancy. It should be remembered RDW values may change as a results of iron deficiency anemia, chronic inflammatory diseases (UC and CD), malnutrition or even germline mutation that predisposes to colorectal cancer.

One of the limitation of ours and others authors’ studies is that they are not adjusted for these confounding factors. Therefore it cannot be concluded that RDW is an independent risk factor of poor survival in colorectal cancer patents.

Also our multivariate Cox regression analysis confirms this statement. In our study, high and low RDW values adjusted for age, gender, tumor location, intent of surgery and TNM stage, were not found to be an independent predictor of prognosis either for overall or cancer related survival.

In conclusion, we believe that RDW represents an important prognostic factor of overall survival.

However, even though it would appear to have some prognostic value in colorectal cancer, the assumption that H-RDW values correlate with a more extensive or aggressive disease should not be made. It is possible that factors that promote, or are a consequence of CRC, could cause changes to RDW values.

Further highly powered studies are needed to elucidate the role of RDW in each specific malignancy. The presence of many confounding factors in these patient’s cohorts may render the definition of the prognostic value of RDW very difficult to achieve.

## Methods

### Patients and eligibility criteria

All patients undergoing surgery for CRC at the Division of General and Hepatobiliary Surgery, University of Verona Hospital Trust, between January 2005 and December 2016 were evaluated for study feasibility. Inclusion criteria were: elective surgery for pathology-proven CRC, age 18 or older, availability of pre-operative and up to 24 months follow-up RDW values.

Patients with evidence of infections or other inflammatory conditions during the pre-operative evaluation were excluded. Patients who underwent surgery within an emergency setting (obstruction, bleeding, perforation) were also excluded.

All methods used in this study were performed in accordance with the relevant ethical guidelines and regulations of the University Hospital of Verona, where the investigation was carried out. The study was approved by the institutional review board and ethic committee of the University of Verona Hospital (ID number: 42763 - CRINF-1034 CESC). Informed consent was obtained from all patients enrolled in the study.

### Preoperative work-up, extent of surgery and histopathological staging

Preoperative work-up, extent of surgery and histopathology staging for CRCs have previously been reported by our group^2^.

All patients were staged with colonoscopy, chest-abdomen and pelvis computed tomography (CT) and carcinoembryonic antigen (CEA). Additional imaging studies like magnetic resonance imaging (MRI) and positron emission tomography (PET-TC) were performed when indicated.

The complete excision of the tumour burden was the main outcome of surgery. Standard open or laparoscopic CRC resections with ligation of vessels at their origin were usually performed to harvest an adequate number of lymph nodes. The mean number of analysed nodes was 20.5 (SD, 10.3); cases with 12 or more analysed nodes were 84.4%.

Pathology specimens were reported according to the 7^th^ Edition of the American Joint Committee on Cancer (AJCC) guidelines and the Union International Contre Le Cancer (UICC) criteria.

### Preoperative assessment of laboratory data

Red cell distribution width (RDW) was measured in venous blood within 2 weeks prior to the date of surgery. Blood samples were drawn by an expert phlebotomist in vacuum blood tubes containing K_2_-EDTA (Terumo Europe NV, Leuven, Belgium). The complete blood cell count (CBC) was performed using Advia 2120 (Siemens Healthcare Diagnostics, Tarrytown NY, USA). The quality and comparability of test results was validated by data of both internal quality control (IQC) and external quality assessment (EQA). The same blood analyser was used throughout the study period and the local reference range was 11.5% to 15%.

### Follow-up and statistical analysis

All clinical and pathological data were retrospectively collected and stored in a digital database. Demographic, clinical, surgical and pathology variables were analysed.

On preliminary analysis, preoperative RDW was found to be normally distributed. The optimal cut-off values for RDW as dichotomous predictor of survival was chosen after considering i) conventional receiver operating characteristics (ROC) curve analysis using death as the outcome; ii) Kaplan-Meier curves and proportional hazards regression model with cut-off increased in progressive steps and results recalculated at each step in order to identifying the threshold associated with the greatest separation of curves with the lowest p value; iii) evaluation of cut-offs proposed by previous literature. Accordingly, two groups of patients were considered: those with a value equal or lower than the median (L-RDW: ≤14.1%) and those with a value higher than the median (H-RDW: >14.1).

The significance of differences was evaluated with chi square test or Fisher’s exact test for categorical data (using RDW as dichotomous variable), and Student’s t-test for continuous variables. The H and L-RDW patients overall and cancer-related survival were analysed with Kaplan-Meier curves and compared with the log-rank test. The time of survival was measured between the date of surgery and the date of the most recent follow-up or death. Multivariable analysis for overall survival and cancer-related survival was performed using Cox regression model by considering RDW values above or below the median and adjusting for the following risk factors: age (>median vs. ≤median), gender (male vs. female), tumour location (rectum vs. colon), intent of surgery (R1-2/No resection vs. R0 resection), and AJCC/UICC TNM stage (stage II, stage III and stage IV vs. stage 0-I). A p value < 0.05 was considered to be statistically significant.

Statistical analysis was performed using SPSS software version 21.0 version (IBM Corporation, Armonk, NY) and STATA software (Stata Corporation, 2011, MP-Parallel Edition).
